# Association of Breakfast Consumption Frequency with Depression and Anxiety Symptoms Among School Students: A Cross-Sectional Study in Eastern China

**DOI:** 10.3390/nu17071271

**Published:** 2025-04-05

**Authors:** Hao Wang, Yunqi Guan, Huaidong Du, Pinyuan Dai, Jieming Zhong, Min Yu, Na Li

**Affiliations:** 1 Department of Non-Communicable Diseases Control and Prevention, Zhejiang Provincial Center for Disease Control and Prevention, Hangzhou 310051, China; wanghao7710@163.com (H.W.); yqguan@cdc.zj.cn (Y.G.); pydai@cdc.zj.cn (P.D.); jmzhong@cdc.zj.cn (J.Z.);; 2 Clinical Trial Service Unit & Epidemiological Studies Unit (CTSU), Nuffield Department of Population Health, University of Oxford, Oxford OX3 7LF, UK; huaidong.du@ndph.ox.ac.uk

**Keywords:** breakfast consumption, depression symptoms, anxiety symptoms, adolescents

## Abstract

**Objective**: This study aimed to explore the relationship between breakfast consumption frequency and both depression and anxiety symptoms among middle and high school students in Eastern China. **Methods**: In this school-based cross-sectional study, 27,001 middle and high school students were investigated in 2022. Multivariable logistic regression models were used to examine the relationship between breakfast consumption frequency and both depression and anxiety symptoms. **Results**: The percentages of students who consumed breakfast daily, 6 days/week, 4–5 days/week, and ≤3 days/week were 71.0% (95%CI: 69.9–72.2), 8.3% (95%CI: 7.8–8.6), 11.9% (95%CI: 11.2–12.6), and 8.8% (95%CI: 8.2–9.5), respectively. After adjusting for socio-demographic and lifestyle factors, academic performance, self-reported health, and bullying victimization, compared to those consuming breakfast daily, the odds ratios (95%CI) for depression symptoms were 1.32 (1.15–1.52) for those consuming breakfast 6 days/week, 1.66 (1.49–1.84) for those consuming breakfast 4–5 days/week, and 1.74 (1.54–1.97) for those consuming breakfast ≤3 days/week, respectively (*p* < 0.001). The corresponding figures for anxiety symptoms were 1.31 (1.14–1.51), 1.35 (1.20–1.52), and 1.43 (1.23–1.66), respectively (*p* < 0.001). **Conclusions**: Breakfast skipping is common among middle and high school students in Eastern China. The frequency of breakfast consumption is inversely associated with both depression symptoms and anxiety symptoms among adolescents.

## 1. Introduction

Mental disorders are responsible for numerous and increasing health burdens worldwide. The Global Burden of Diseases 2019 indicated that the global age-standardized rates of disability-adjusted life-years (DALYs) of mental disorders increased from 803.8 per 100,000 in 1990 to 833.2 per 100,000 in 2019 among children and adolescents (i.e., <20 years old) [1]. During the same period, the corresponding figures for anxiety disorders increased from 218.5 per 100,000 to 222.2 per 100,000, and depressive disorders increased from 153.6 per 100,000 to 170.0 per 100,000, respectively [1]. In China, mental disorders are among the leading causes of years lived with disability (YLDs) and the third leading cause of DALYs in children and adolescents in 2021 [2]. Of the total 30.8 million children and adolescents with mental disorders in China, 9.0 million were diagnosed with anxiety disorders, and 1.5 million were diagnosed with depression disorders [2].

Breakfast is widely recognized as the most crucial meal of the day [3], and a healthy breakfast could contribute approximately 20–35% of the total daily energy [4]. The early establishment of healthy breakfast habits may persist into adulthood [5,6]. However, the percentage of daily breakfast consumption among Chinese primary and middle school students declined sharply from 86.5% in 1992 to 62.4% in 2017 [7]. Similar trends were observed globally, with US high school students’ daily breakfast intake dropping from 38.0% to 27.0% between 2013 and 2023 [8] and comparable decreases reported across 15 European countries [9].

Previous studies have documented that breakfast skipping is not only associated with school absenteeism, poor academic performance, and poor health-related behaviors [10,11] but is also associated with elevated risks of obesity [12,13], hypertension [14], cardiovascular disease [15,16], stroke [16], chronic kidney disease [17,18], and diabetes [19,20]. However, the existing literature on the association of breakfast skipping with depression symptoms remains inconclusive. Some studies documented positive associations [21,22,23], while others observed null associations [24,25]. Similarly, the association of breakfast skipping with anxiety symptoms remains conflicting [22,26,27], with limited research specifically investigating this association [28]. To address these knowledge gaps, this study aims to systematically evaluate the associations between breakfast consumption and both depression and anxiety symptoms among students in Eastern China.

## 2. Materials and Methods

### 2.1. Survey Design and Participants

**Figure 1:**
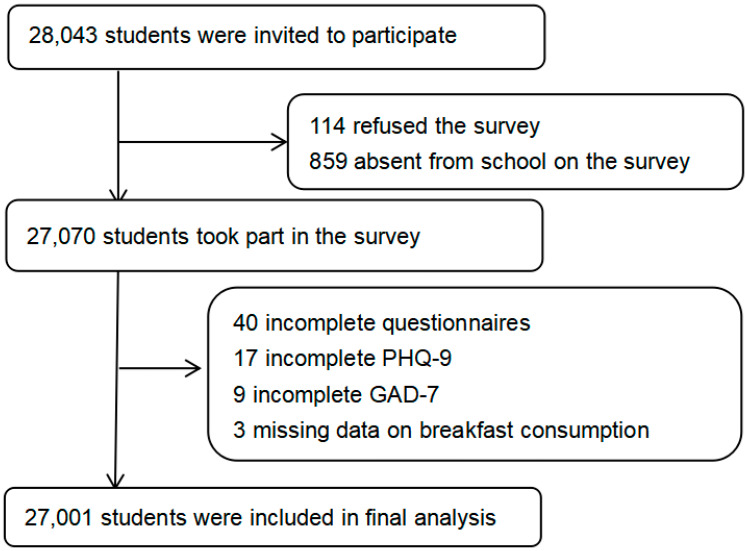
Flowchart of participants included in final analysis.

A three-stage cluster sampling design was conducted to obtain a representative sample of students in grades 7–12. In the first sampling stage, 30 counties/districts were sampled randomly from all 90 counties/districts. Details on the location of 30 counties/districts are available in the Supplementary Materials (Figure S1). In the second sampling stage, 11 middle school classes, 6 academic high school classes, and 6 vocational high school classes were selected randomly within each selected county/district. In the third sampling stage, all students in the identified classes were invited to participate. The inclusion criteria for students were as follows: not having serious health conditions or illnesses that prevent students from participating, including intellectual disability, etc.; signed consents from students and their legal guardians. Students were excluded if any of the following conditions were identified: having serious health conditions or illness; refusal to participate; or absence from school on the survey day. Out of a total of 28,043 eligible students, 114 students refused to participate and 859 were absent from school on the survey day, achieving a 96.5% response rate (27,070 participants). After excluding 69 students due to incomplete questionnaires, 27,001 students were included in the final analysis (Figure 1).

The survey questionnaire was adapted from validated questionnaires, including the US Youth Risk Behavior Survey and the Global School-based Student Health Survey. The field survey was conducted by well-trained staff following standardized protocols. To ensure data integrity, multiple quality control measures were implemented. Questionnaires were completed anonymously during school hours in classroom settings. Physical distancing (≥1 m between participants) was enforced to ensure privacy.

### 2.2. Sample Size Calculation

The formula for sample size calculation is the following: *N* = *deff* × µ^2^ × *p ×* (1 − *p*)/*d*^2^. Means and 95% confidence intervals (CIs; 2-sided for *u* = 1.96) were determined; the prevalence of daily breakfast consumption in China (72%) was used as a measure of probability (*p*) [29]; the design effect (*deff*) value was set at 3; and the relative error was *d* = *r* × 72%, *r* = 5%. Based on the parameters above, the sample size for each stratum was estimated to be 1793. Because there were 12 strata (Areas: urban and rural. Sex: boy and girl. School types: middle schools, academic high schools, and vocational high schools), and assuming a potential non-response rate of 20%, the final sample size was estimated to be 26,895.

### 2.3. Measures

#### 2.3.1. Breakfast Consumption Assessment

Breakfast consumption frequency was measured with the following question: “During the past 7 days, on how many days did you eat breakfast? (Response options: “None”, “1 day”, “2 days”, “3 days”, “4 days”, “5 days”, “6 days”, and “7 days”)” [30]. Breakfast skippers were defined as individuals who reported skipping breakfast on one or more days per week, and non-skippers were defined as individuals who reported consuming breakfast daily.

#### 2.3.2. Depression Symptoms Assessment

Depression symptoms were assessed using the validated 9-item Patient Health Questionnaire (PHQ-9). Each item is rated from 0 (not at all) to 3 (nearly every day). Cumulative scores range from 0 to 27, with elevated scores indicating greater severity of depressive symptoms. Depression was defined as a cumulative score ≥ 10 in the current study, with a specificity of 85% and sensitivity of 88% [31].

#### 2.3.3. Anxiety Symptoms Assessment

Anxiety symptoms were assessed using the generalized anxiety disorder scale (GAD-7), comprising a total of 7 items. Each item is rated from 0 (not at all) to 3 (nearly every day). Cumulative scores range from 0 to 21, with higher scores indicating higher GAD levels. A cumulative score of 10 or more is considered GAD [32,33]. The GAD-7 scale has good validity and reliability [34].

### 2.4. Covariates

Covariates in the present study comprise age, gender, region, types of school, parental education, parental marital status, family economic status, cigarette use, alcohol use, physical activity, academic performance, self-reported health, sleep duration, and bullying victimization (Table 1). Cigarette smoking was defined as smoking cigarettes at least one day during the past 30-day period. Alcohol drinking was defined as drinking alcohol at least one day during the past 30-day period. Being physically active was defined as engaging in moderate-to-vigorous physical activity (MVPA) for ≥60 min daily. Height was assessed through the question: “How tall are you without your shoe on?”. Weight was assessed through the question: “How much do you weigh without your shoes on?” Body mass index (BMI) was calculated as self-reported weight in kilograms divided by the square of self-reported height in meters.

### 2.5. Statistical Analysis

Data quality was ensured through dual-entry verification using Epidata 3.1 software with built-in validation checks. All analyses were adjusted for the complex sampling design using appropriate sample weights. Continuous variables were presented as the mean ±standard deviation. Categorical variables were summarized as a percentage and 95% confidence intervals (CIs). Rao–Scott χ^2^ tests were conducted to test for differences in percentages. The baseline characteristics of participants between different groups of breakfast consumption frequency were compared using either general linear regression (for continuous variables) or logistic regression (for categorical variables), adjusting for age and sex. Multivariable logistic regression analyses were utilized to appraise the association of breakfast consumption frequency with depression symptoms and anxiety symptoms. In Model 1, ORs were adjusted for age (continuous), gender (boys or girls), region (urban or rural), types of school (middle school, academic high school, and vocational high school), parental education (middle school or below, high school, and college or above), parental marital status (married or others), and family economic status (very poor/poor, fair, very rich/rich). Model 2 was additionally adjusted for cigarette smoking (yes or no), alcohol drinking (yes or no), being physically active (none, 1–2 days/week, 3–5 days/week, and 6–7 days/week), academic performance (excellent, middle, and poor), sleep duration (continuous), and self-reported health (very good/good, fair, very bad/bad, and unknown). Model 3 was additionally adjusted for bullying victimization (yes or no). Sensitivity analyses were additionally adjusted for BMI (continuous). All statistical analyses were performed using SAS software Version 9.4 (SAS Institute, Cary, NC, USA). Statistical significance was determined as a two-tailed *p*-value < 0.05.

## 3. Results

### 3.1. General Characteristics of Participants

Sample descriptives are provided in Table 1. The final analytical sample comprised 13,930 boys and 13,071 girls. The mean age was 15.6 ± 1.7 years. The student numbers from middle schools, academic high schools, and vocational high schools were 12,775 (47.3%), 7376 (27.3%), and 6870 (25.4%), respectively. In total, 12.4% lived in non-intact family structures. A total of 8.9% reported high/very high family economic status. The fathers of 18.8% of students were educated to college level or higher, and the mothers of 18.1% of students were educated to college level or higher. In total, 45.0% of students were physically active ≤ 2 days/week. A total of 19.6% self-rated their academic performance as excellent. A total of 53.6% perceived their health status as good/very good. A total of 3.9% of students were cigarette smokers, and 16.0% were alcohol drinkers. The mean sleep duration was 8.0 ± 2.0 h/day. In total, 30.0% reported bullying victimization during the preceding 30 days, 22.4% reported depression symptoms, and 14.2% reported anxiety symptoms.

### 3.2. Frequency of Breakfast Consumption

In total, 71.0% (95%CI: 69.9–72.2) of students consumed breakfast daily. In total, 8.3% (95%CI: 7.8–8.6) consumed breakfast 6 days per week. In total, 11.9% (95%CI: 11.2–12.6) consumed breakfast 4–5 days per week. In total, 8.8% (95%CI: 8.2–9.5) consumed breakfast ≤ 3 days per week. The percentage of non-skippers among students aged ≤13 years, 14–15 years, and ≥16 years were 74.6% (95%CI: 72.5–76.6), 70.7% (95%CI: 68.9–72.5), and 69.5% (95%CI: 67.8–71.1), respectively (*p* < 0.001). The percentage of non-skippers among boys (73.7%, 95%CI: 72.3–75.2) was higher than girls (68.1%, 95%CI: 66.7–69.4) (*p* < 0.001). The percentage of non-skippers in urban areas (73.2%, 95%CI: 71.4–75.0) was higher than in rural areas (69.9%, 95%CI: 68.4–71.4) (*p* = 0.007). The corresponding figures for students attending middle school, academic high school, and vocational high school were 72.6% (95%CI: 71.0–74.2), 74.7% (95%CI: 72.6–76.8), and 63.0% (95%CI: 61.1–65.0), respectively (*p* < 0.001) (Table 2).

### 3.3. Association Between Breakfast Consumption Frequency and Depression and Anxiety Symptoms

After adjusting for socio-demographic factors, lifestyle factors, academic performance, self-reported health, and bullying victimization, compared to those consuming breakfast daily, the odds ratios (95%CI) for depression symptoms were 1.33 (1.15–1.52) for those consuming breakfast 6 days/week, 1.66 (1.49–1.84) for those consuming breakfast 4–5 days/week, and 1.74 (1.54–1.97) for those consuming breakfast ≤3 days/week, respectively (*p*-trend < 0.001) (Table 3).

After adjusting for socio-demographic factors, lifestyle factors, academic performance, self-reported health, and bullying victimization, compared to those consuming breakfast daily, the odds ratios (95%CI) for anxiety symptoms were 1.31 (1.13–1.51) for those consuming breakfast 6 days/week, 1.35 (1.20–1.52) for those consuming breakfast 4–5 days/week, and 1.43 (1.23–1.66) for those consuming breakfast ≤3 days/week, respectively (*p*-trend < 0.001) (Table 4).

### 3.4. Sensitivity Analyses

In sensitivity analyses, after excluding participants with missing values of self-reported height or self-reported weight (*N* = 270) with BMI < 12.0 kg/m^2^ (*N* = 58) and BMI ≥ 35.0 kg/m^2^ (*N* = 138), 26,535 students were included in the analyses. After further adjustment for BMI, there was no marked change in the association of breakfast consumption frequency with both depression symptoms and anxiety symptoms (Table S1 is included in the Supplementary Materials).

## 4. Discussion

In this provincially representative study of Chinese adolescents, the updated results and patterns of breakfast skipping are provided. Furthermore, the associations of breakfast consumption frequency with both depression symptoms and anxiety symptoms were elucidated, and a dose–response relationship was observed between breakfast consumption frequency and mental health risks.

### 4.1. Prevalence of Breakfast Skipping

Due to different measures and definitions of breakfast skipping, direct comparisons between studies are difficult. For example, some studies inquired about the frequency of breakfast consumption during school days (i.e., from Monday to Friday) [35], while others inquired about the frequency of breakfast consumption during the past 7 days [30,36,37]. Response options in some studies were “rarely”, “sometimes”, and “often” [11,38] but ranged from “0 days” to “7 days” in other studies [30,39,40]. In the present study, 71.0% of students ate breakfast daily, which was higher than most of the previous studies. For example, a US study of high school students in 2023 indicated that 27.4% of students ate breakfast daily [30]. An Australian study of 32,498 students in grades 8–12 in 2019 indicated that 42.6% of students ate breakfast daily [41]. A systematic review of 286,804 children and adolescents living in 33 countries observed that the prevalence of breakfast skipping ranged from 10 to 30% [42]. One representative Chinese study of 120,285 children and adolescents aged 9–18 years demonstrated that 72.6% ate breakfast daily in 2019 [29]. Another representative Chinese study of 15,415 middle and high school students indicated that 62.4% ate breakfast daily in 2017 [7]. In total, 66.6% of primary and high school students ate breakfast daily in Southwest China [26]. In contrast, a Japanese study of 1510 junior high school students aged 13–14 years documented that 83.6% ate breakfast daily [43], which is higher than the current study. It is noteworthy that although breakfast-eating behavior is more prevalent in the current study population than in other populations, nearly 30% of students still skip breakfast at least once weekly in the current study. Middle and high school students are in a transition from childhood to adulthood, and daily breakfast consumption is crucial for their physical growth and brain development. Hence, effective intervention to improve breakfast consumption among adolescents needs to be reinforced in China. In line with previous studies [29,35,41,43], skipping breakfast was more prevalent among girls than boys. Consistent with the previous studies [35,44], skipping breakfast was more common among older students.

### 4.2. Association Between Breakfast Consumption Frequency and Depression Symptoms

A study of 21,972 university students from 28 countries documented that the odds ratio for depression among students who never or rarely consumed breakfast was 2.13 (95%CI: 2.03–2.69) in comparison with those who ate breakfast daily [11]. A prospective study of 757 Chinese university students revealed that compared to students consuming breakfast 6–7 days/week, the odds ratios (95%CI) for depression symptoms were 2.05 (1.20–3.49) for those consuming breakfast 2–5 days/week, and 2.72 (0.94–7.87) for those consuming breakfast ≤1 days/week, respectively [39]. A cross-sectional study of 11,887 Chinese students aged 11–19 years old indicated that after adjusting for all confounders, students who sometimes or always skipped breakfast were associated with an increased risk of depression in comparison to those who reported consuming breakfast daily (OR = 2.56, 95%CI: 2.24–2.93) [23]. A 2022 meta-analysis of six studies demonstrated that breakfast skipping was positively related to depression among adolescents (OR = 1.36, 95%CI: 1.30–1.43) [28]. In the present study, the frequency of breakfast consumption was negatively associated with the presence of depressive symptoms, which is in line with those studies mentioned above.

### 4.3. Association Between Breakfast Consumption Frequency and Anxiety Symptoms

Contrary to the present study, an Australian study of 751 university students documented that breakfast skipping is not associated with anxiety symptoms [27]. One Chinese study of 23,005 primary and high school students indicated that compared to students eating breakfast daily, the odds ratios (95%CI) for anxiety symptoms were 1.20 (1.09–1.32) for those eating breakfast 5–6 days/week, 1.15 (1.01–1.30) for those eating breakfast 3–4 days/week, 1.24 (1.05–1.48) for those not eating breakfast 1–2 days/week, and 1.09 (0.79–1.50) for those who never eat breakfast, respectively [26]. Another Chinese study of 384 college students revealed breakfast skippers had a 2.33 times higher probability of anxiety than non-skippers (OR = 2.33, 95%CI: 1.44–3.77) [38]. In addition, a meta-analysis of three studies, including 16,544 individuals aged ≥6 years, demonstrated that a positive association between breakfast skipping and anxiety was observed only among adolescents (OR = 1.54, 95%CI: 1.47–1.62), not among adults (OR = 0.92, 95%CI: 0.49–1.34) [28].

Although mechanisms underlying the potential beneficial effect of breakfast consumption on mental health have not yet been fully elucidated, several possible hypotheses have been proposed. First, breakfast skipping might lead to obesity [13,45], and obesity is positively associated with the risk of mental health [46,47]. The traditional Chinese breakfast consists of whole grains (e.g., congee, noodles), vegetables, dairy products, eggs, etc. Whole grains exert protective effects on mental health [48]. Eggs are rich in bioactive compounds, including choline and carotenoids, which may have the potential to help mitigate the risk of mental health [49,50]. Vegetable intake, abundant in various minerals and vitamins, such as magnesium, zinc, selenium, and vitamin B12, is inversely associated with the risk of depression and anxiety [51,52].

### 4.4. Strengths and Limitations

The strengths of this study consist of a large-scale sample size, a random sampling design, a high response rate, and a standardized procedure. However, several limitations should be acknowledged. First, the nature of this cross-sectional study prohibits the establishment of a temporal relationship between breakfast consumption and depression and anxiety. Longitudinal studies are needed to confirm this relationship. Second, the present study failed to collect information on breakfast content and the reasons behind breakfast skipping, which could help policymakers better and, more precisely, improve breakfast skipping. A higher caloric ratio of carbohydrate intake was reported to be associated with increased depressive symptoms in general adults [53]. Third, although analyses were adjusted for most established potential confounding factors, residual confounding might still exist. The home environment and family medical and psychiatric history, which may influence the development of children’s mental health [54,55], were not included in the current study.

## 5. Conclusions

Despite the aforementioned limitations, our study sheds light on the association between breakfast consumption and both depression and anxiety symptoms among middle and high school students in Eastern China. We found that breakfast skipping appeared prevalent and was positively associated with both depression and anxiety symptoms. Preventive measures for breakfast skipping among adolescents need to be further reinforced in Eastern China, and efforts to prevent adolescent mental health may need to address breakfast skipping.

## Figures and Tables

**Table 1:** Participant characteristics by frequency of breakfast consumption ^a^.

Characteristics	Total (*N* = 27,001)	Frequency of Breakfast Consumption				*p*-Trend
		≤3 Days	4–5 Days	6 Days	7 Days	
		(*N* = 2452)	(*N* = 3346)	(*N* = 2222)	(*N* = 18,981)	
Mean age (years)	15.6 ± 1.7	15.6 ± 1.7	15.8 ± 1.6	15.9 ± 1.7	15.6 ± 1.7	0.206
Girls (%)	47.4	47.0	54.3	55.1	45.4	<0.001
Urban (%)	35.4	34.8	31.8	31.8	36.4	<0.001
Middle school (%)	51.8	54.5	48.3	44.2	53.0	0.431
Living in non-intact families (%)	12.4	19.0	16.0	14.1	10.8	<0.001
Father educated to college level or high (%)	18.8	13.8	13.0	16.0	20.7	<0.001
Mother educated to college level or high (%)	18.1	13.9	11.6	15.5	20.0	<0.001
High family income (%)	8.9	8.4	7.1	7.9	9.3	0.001
Physically active ≤ 2 d/week (%)	45.0	49.7	48.4	47.5	43.6	<0.001
Excellent academic performance (%)	19.6	14.1	12.9	16.6	21.8	<0.001
Cigarette smoking (%)	3.9	8.3	7.0	3.9	2.8	<0.001
Alcohol drinking (%)	16.0	25.8	24.7	19.4	13.0	<0.001
Good self-reported health (%)	53.6	44.7	41.6	43.3	57.9	<0.001
Sleep duration (hours)	8.0 ± 2.0	7.9 ± 2.6	7.9 ± 2.2	7.8 ± 1.5	8.1 ± 1.9	<0.001
Bullying victimization (%)	30.0	39.8	37.4	35.3	26.9	<0.001
Depression symptoms (%)	22.4	36.2	34.4	28.6	18.0	<0.001
Anxiety symptoms (%)	14.2	22.3	20.6	19.0	11.6	<0.001

^a^ Based on the weighted data. Continuous variables are presented as the mean ± SD, and categorical variables are presented as percentages. Characteristics of participants between different groups were compared using either general linear regression (for continuous variables) or logistic regression (for categorical variables), adjusting for age and sex.

**Table 2:** Weighted percentage of frequency of breakfast consumption ^a^.

	Frequency of Breakfast Consumption			
	≤3 Days	4–5 Days	6 Days	7 Days
	(*N* = 2452)	(*N* = 3346)	(*N* = 2222)	(*N* = 18,981)
Age range (years)				
≤13	8.8 (7.8–9.9)	9.9 (8.7–11.0)	6.7 (5.8–7.7)	74.6 (72.5–76.6)
14–15	9.6 (8.6–10.7)	12.3 (11.3–13.4)	7.4 (6.7–7.9)	70.7 (68.9–72.5)
≥16	8.2 (7.2–9.2)	12.7 (11.7–13.7)	9.6 (8.9–10.3)	69.5 (67.8–71.1)
Gender				
Boys	8.9 (8.1–9.7)	10.4 (9.6–11.2)	7.0 (6.5–7.5)	73.7 (72.3–75.2)
Girls	8.8 (7.9–9.6)	13.7 (12.7–14.6)	9.4 (8.9–10.2)	68.1 (66.7–69.4)
Area				
Urban	8.7 (7.6–9.8)	10.7 (9.7–11.8)	7.4 (6.7–8.0)	73.2 (71.4–75.0)
Rural	8.9 (8.1–9.7)	12.6 (11.6–13.5)	8.6 (8.1–9.2)	69.9 (68.4–71.4)
Types of school				
Middle school	9.3 (8.5–10.1)	11.1 (10.2–12.0)	7.0 (6.4–7.5)	72.6 (71.0–74.2)
Academic high school	7.3 (5.7–8.8)	8.0 (7.1–8.9)	10.0 (9.0–11.1)	74.7 (72.6–76.8)
Vocational high school	9.6 (8.3–10.9)	18.5 (16.9–20.0)	8.9 (8.1–9.6)	63.0 (61.1–65.0)

^a^ Data are presented as percentages (95%CI). Rao–Scott χ^2^ tests were conducted to test for differences across groups.

**Table 3:** Adjusted odds ratios of depression symptoms associated with frequency of breakfast consumption ^a^.

	Frequency of Breakfast Consumption				*p*-Trend
	7 Days	6 Days	4–5 Days	≤3 Days	
	(*N* = 18,981)	(*N* = 2222)	(*N* = 3346)	(*N* = 2452)	
Total					
Model 1	1.00 (Ref)	1.70 (1.50–1.92)	2.31 (2.11–2.53)	2.50 (2.25–2.79)	<0.001
Model 2	1.00 (Ref)	1.39 (1.21–1.60)	1.72 (1.55–1.91)	1.84 (1.63–2.07)	<0.001
Model 3	1.00 (Ref)	1.33 (1.15–1.52)	1.66 (1.49–1.84)	1.74 (1.54–1.97)	<0.001
Boys					
Model 1	1.00 (Ref)	1.59 (1.32–1.91)	2.11 (1.79–2.49)	2.34 (2.00–2.74)	<0.001
Model 2	1.00 (Ref)	1.30 (1.06–1.59)	1.62 (1.36–1.93)	1.82 (1.53–2.17)	<0.001
Model 3	1.00 (Ref)	1.19 (0.97–1.45)	1.58 (1.32–1.89)	1.72 (1.44–2.06)	<0.001
Girls					
Model 1	1.00 (Ref)	1.78 (1.52–2.10)	2.46 (2.20–2.76)	2.64 (2.30–3.03)	<0.001
Model 2	1.00 (Ref)	1.46 (1.23–1.75)	1.76 (1.55–2.01)	1.82 (1.53–2.17)	<0.001
Model 3	1.00 (Ref)	1.43 (1.20–1.71)	1.70 (1.48–1.95)	1.73 (1.45–2.07)	<0.001

^a^ Data are presented as OR (95%CI) estimated by logistic regression models. Model 1: odds ratios were adjusted for age, gender, region, types of school, parental education, parental marital status, and family income. Model 2: odds ratios were additionally adjusted for cigarette smoking, alcohol drinking, physical activity, academic performance, sleep duration, and self-reported health. Model 3: odds ratios were additionally adjusted for bullying victimization. Abbreviations: Ref, reference.

**Table 4:** Adjusted odds ratios of anxiety symptoms associated with frequency of breakfast consumption ^a^.

	Frequency of Breakfast Consumption				*p*-Trend
	7 Days	6 Days	4–5 Days	≤3 Days	
	(*N* = 18,981)	(*N* = 2222)	(*N* = 3346)	(*N* = 2452)	
Total					
Model 1	1.00 (Ref)	1.67 (1.46–1.90)	1.92 (1.73–2.13)	2.13 (1.86–2.44)	<0.001
Model 2	1.00 (Ref)	1.38 (1.19–1.59)	1.41 (1.26–1.58)	1.53 (1.32–1.78)	<0.001
Model 3	1.00 (Ref)	1.31 (1.14–1.51)	1.35 (1.20–1.52)	1.43 (1.23–1.66)	<0.001
Boys					
Model 1	1.00 (Ref)	1.70 (1.37–2.09)	1.73 (1.44–2.08)	2.11 (1.76–2.53)	<0.001
Model 2	1.00 (Ref)	1.40 (1.11–1.77)	1.32 (1.08–1.61)	1.62 (1.32–1.99)	<0.001
Model 3	1.00 (Ref)	1.28 (1.03–1.60)	1.28 (1.04–1.56)	1.52 (1.23–1.87)	<0.001
Girls					
Model 1	1.00 (Ref)	1.65(1.39–1.98)	2.02 (1.77–2.31)	2.12 (1.80–2.49)	<0.001
Model 2	1.00 (Ref)	1.35(1.11–1.65)	1.45 (1.25–1.67)	1.46 (1.21–1.75)	<0.001
Model 3	1.00 (Ref)	1.32 (1.09–1.60)	1.38 (1.18–1.61)	1.37 (1.14–1.64)	<0.001

^a^ Data are presented as OR (95%CI) estimated by logistic regression models. Model 1: odds ratios were adjusted for age, gender, region, types of school, parental education, parental marital status, and family income. Model 2: odds ratios were additionally adjusted for cigarette smoking, alcohol drinking, physical activity, academic performance, sleep duration, and self-reported health. Model 3: odds ratios were additionally adjusted for bullying victimization. Abbreviations: Ref, reference.

## Data Availability

The datasets used and/or analyzed during the current study are available from H.W. on reasonable request. The data are not publicly available due to privacy restrictions.

## Supplementary Materials

The following supporting information can be downloaded athttps://www.mdpi.com/article/10.3390/nu17071271/s1, Figure S1: Distribution of 30 survey counties/districts. Table S1: Adjusted odds ratios of mental health associated with frequency of breakfast consumption after further adjustment for BMI.

## Abbreviations

DALYs	Disability-Adjusted Life Years
YLDs	Years Lived with Disability
US	United States
PHQ-9	9-item Patient Health Questionnaire
GAD-7	7-item Generalized Anxiety Disorder
MVPA	Moderate-to-vigorous physical activity
CI	Confidence intervals
OR_S_	Odds ratios
