# Supplementary material for: Association of Breakfast Consumption Frequency with Depression and Anxiety Symptoms Among School Students: A Cross-Sectional Study in Eastern China
Source: Nutrients. 2025 Apr 5;17(7):1271. doi: 10.3390/nu17071271 (PMC11990333; doi:10.3390/nu17071271)
Supplement: Supplementary file 1 [file nutrients-17-01271-s001.zip › nutrients-3560958-supplementary.pdf]

SUPPLEMENTARY MATERIAL

Figure S1: Distribution of 30 survey counties/districts. **Table S1:** Adjusted odds ratios of mental health associated with frequency of breakfast consumption after further adjustment for BMI.

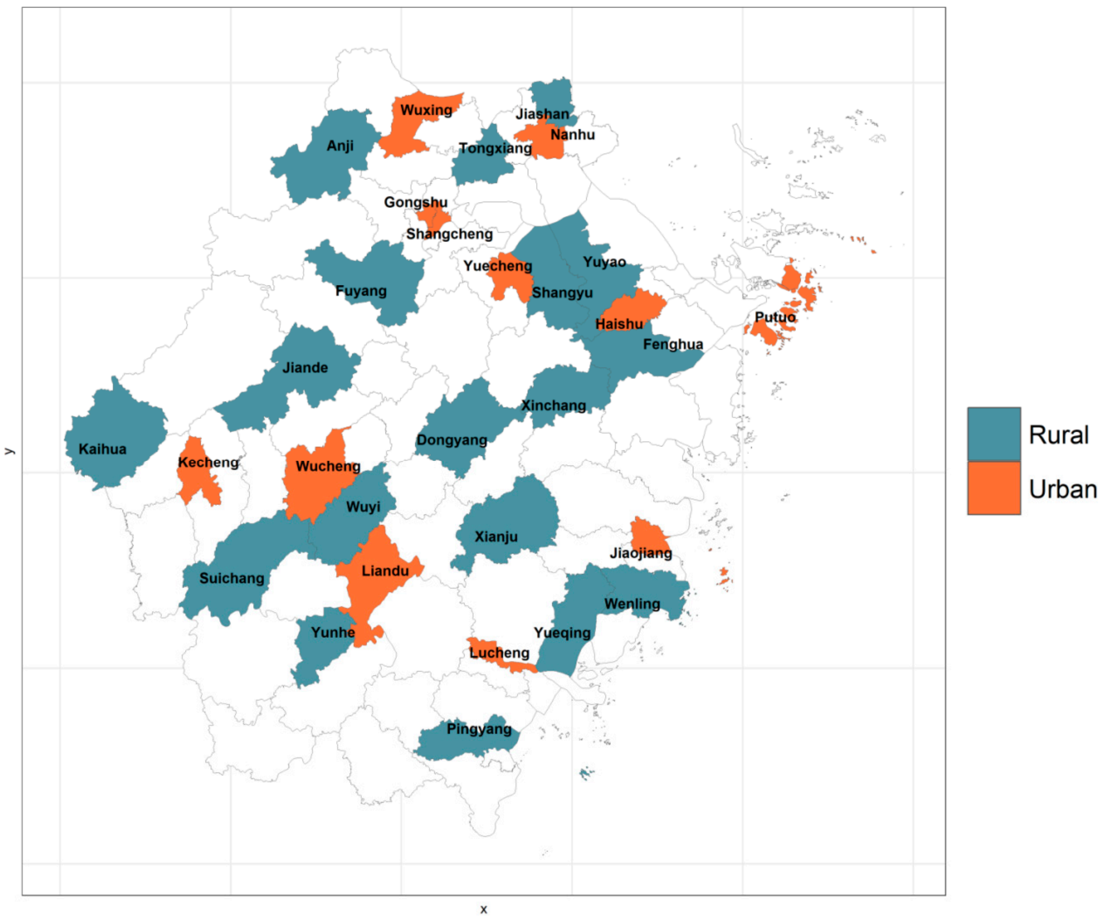

Figure S1: Distribution of 30 survey counties/districts

Table S1. Odds ratios of mental health associated with frequency of breakfast consumption after further adjustment for BMI [a].

	Frequency of breakfast consumption				*p*-Trend
	**7 days (*N* = 18,685)**	**6 days (*N* = 2183)**	**4-5 days (*N* = 3280)**	**≤ 3days (*N* = 2387)**	
**Depression symptoms**					
Total	1.00 (Ref)	1.32 (1.15-1.52)	1.67 (1.50-1.85)	1.75 (1.55-1.98)	<0.001
Boys	1.00 (Ref)	1.19 (0.97-1.46)	1.59 (1.33-1.90)	1.72 (1.44-2.07)	<0.001
Girls	1.00 (Ref)	1.42 (1.18-1.69)	1.71 (1.49-1.96)	1.76 (1.47-2.10)	<0.001
**Anxiety symptoms**					
Total	1.00 (Ref)	1.31 (1.13-1.51)	1.34 (1.19-1.51)	1.45 (1.25-1.68)	<0.001
Boys	1.00 (Ref)	1.29 (1.02-1.62)	1.28 (1.04-1.57)	1.54 (1.24-1.90)	<0.001
Girls	1.00 (Ref)	1.31 (1.09-1.59)	1.36 (1.16-1.59)	1.39 (1.16-1.66)	<0.001

[a] Data were presented as OR (95%CI) estimated by logistic regression models. Odds ratios were adjusted for age, gender, region, types of school, parental education, parental marital status, family income, cigarette smoking, alcohol drinking, physical activity, academic performance, sleep duration, self-reported health, bullying victimization, and BMI. Abbreviations: Ref, reference.
